# Sensing signal augmentation by flow rate modulation of carrier gas for accurate differentiation of complex odours

**DOI:** 10.1080/14686996.2024.2408212

**Published:** 2024-09-24

**Authors:** Meng-Qun Feng, Tanju Yildirim, Kosuke Minami, Kota Shiba, Genki Yoshikawa

**Affiliations:** a Research Center for Macromolecules and Biomaterials, National Institute for Materials Science (NIMS), Tsukuba, Japan; b Materials Science and Engineering, Graduate School of Pure and Applied Science, University of Tsukuba, Tsukuba, Ibaraki, Japan; c Faculty of Science and Engineering, Southern Cross University, East Lismore, Australia

**Keywords:** Olfactory sensors, gas sensing, e-nose, smell, chemical sensor array, signal augmentation, differentiation, complex odour, gas flow, artificial olfaction

## Abstract

For olfactory sensors, clear differentiation of complex odour samples requires diverse information. To obtain such information, hardware modifications, such as introducing additional channels with different physical/chemical properties, are usually needed. In this study, we present a new approach to augmenting the sensing signals of an olfactory sensor by modulating the flow rate of the carrier gas. The headspace vapour of complex odours is measured using a sensing system of nanomechanical sensor (Membrane-type Surface stress Sensor, MSS). The resulting data set is quantitatively evaluated using the Davies-Bouldin index (DBI) of principal component analysis (PCA). The increasing number of sensing signals obtained at different gas flow rates leads to a decrease in the DBI, achieving better cluster separation between different odours. Such gas flow effects can be attributed to several factors, including the sample evaporation and the equilibrium of the gas-liquid and gas-solid interfaces. Proton-transfer-reaction time-of-flight mass spectrometry (PTR-TOF-MS) experiments reveal that the compositions of odour samples vary with the different gas flow rates. It is demonstrated that a simple technique for modulating gas flow rates can significantly improve the differentiation performance of complex odours, providing an additional degree of freedom in olfactory sensing.

## IMPACT STATEMENT

We present a novel system-based approach to augmenting olfactory sensor signal output by modulating carrier gas flow rates, enhancing odour differentiation performance through experimental, statistical and analytical validation.

## Graphical abstract

**Figure:**
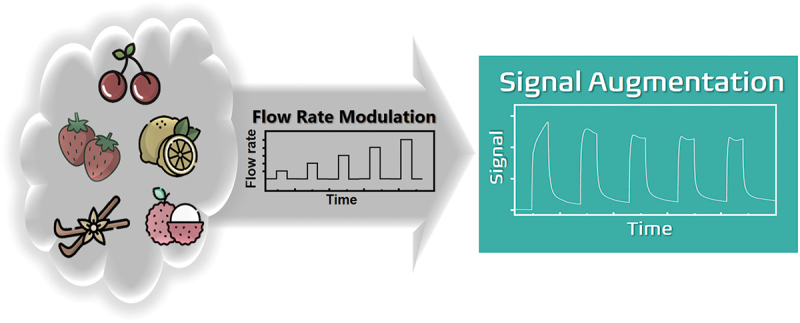

## 1. Introduction

Olfaction, or the sense of smell, is a ubiquitous and intricate system in the natural world, utilised by most living organisms as a rapid means of conveying information of specific gas molecules to the brain. The olfactory system serves as a complex sensory interface, allowing for the detection and discrimination of a wide range of volatile chemicals. The olfactory system in living organisms has inspired the development of olfactory sensors or electronic nose (e-nose) technologies for many applications including medical breath diagnosis [1–8] and smart farming utilising the Internet of Things (IoTs) [1,9–12]. Although these olfactory sensors or e-noses have been developed over recent decades, there are still some technical limitations, such as accurate differentiation of complex odours.

Typical olfactory sensor systems consist of multiple sensors with different physical/chemical properties that enable the analysis of odours through pattern recognition. Some studies have tried to improve the applicability of olfactory sensors via enhancing the diversity of gas-sensitive receptor materials [13,14]. The combination of different receptor materials in a sensor array can effectively improve the diversity of the output signals, providing more information and clearer differentiation of molecules when compared to a single receptor material [11,15–17]. However, preparing multiple receptor materials with different properties is not a trivial task, requiring not only materials design, but also the optimization of conditions for stable coating. Furthermore, adding a measurement channel requires modification of the entire measurement system including electric readout components and peripheral gas flow systems. Therefore, to expand the applicability of olfactory sensors, there is a demand for developing a general method that can provide more information without preparing additional sensor channels with different receptor materials. In recent developments, the methodology of odour discrimination has advanced, particularly in simplifying sensor designs while maintaining discrimination capabilities. Shiba et al. provided an example by using a single channel nanomechanical sensor with designed functional nanoparticles to successfully differentiate between fuel oils [18]. Inspired by this approach, the present study investigates the effects of flow rate modulation in augmenting sensor performance, which could further reduce the reliance on multiple receptor materials for accurate odour differentiation. Such a method would contribute to producing a practical olfactory sensing system with efficient data collection, promoting further development and application of olfactory sensor technologies.

Herein, we propose a system-based approach to augmenting the output signal information from olfactory sensors to extract diverse features. In this approach, the flow rate of carrier gas is varied to modulate the sensing signal. To prove the concept, different odour samples are measured at various flow rates, and the resultant sensing signals are evaluated using the Davies-Bouldin index (DBI) of the resulting principal component analysis (PCA). It is confirmed that the increasing number of sensing signals obtained at different flow rates leads to a decrease in DBI, demonstrating higher performance in discriminating each odour sample with larger cluster separation in PCA. In addition, we measured real-time concentrations of complex odour samples at different flow rates using proton-transfer-reaction time-of-flight mass spectrometry (PTR-TOF-MS). The results indicate that the concentrations of each component vary with the different gas flow rates. Thus, it has been demonstrated and proved that this simple technique of modulating gas flow rates can significantly improve the differentiation performance of complex odours.

## 2. Experimental section

### 2.1. Preparation of sensing chip

#### 2.1.1. MSS sensing system

A nanomechanical Membrane-type Surface stress Sensor (MSS) was utilised as the sensing element (NanoWorld AG, Switzerland) [16,19–21]. The MSS was composed of a thin silicon sensing membrane with a diameter of 300 μm suspended by four narrow bridges with piezoresistors that constitute a full Wheatstone bridge. The sensing membrane was coated with a receptor layer which absorbs gaseous molecules. The receptor layer mechanically deforms upon the sorption of gaseous molecules, inducing electrical sensing signals through the mechanical-electrical transduction at the piezoresistors. Measurements were carried out with a bridge voltage of −0.5 V and a sampling rate of 100 Hz.

#### 2.1.2. Receptor material and inkjet deposition

Polyvinyl alcohol (PVA), poly(vinyl alcohol-co-ethylene) (PVA-co-PE), polyethylene glycol (PEG) and poly(methyl methacrylate) (PMMA) were purchased from Sigma-Aldrich and used as the receptor materials in the experiments. PVA, PVA-co-PE and PEG were dissolved in ultrapure water (MilliQ, Merck MilliPore, Germany) to a concentration of 0.1 g/L. The PVA solution was placed on a hotplate maintained at 100°C until the PVA completely dissolved, while PVA-co-PE and PEG completely dissolved at room temperature. PMMA was dissolved in Dimethylformamide (DMF, Wako Pure Chemical Industries, Ltd) to a concentration of 0.1 g/L. The polymers were deposited onto the surface of an MSS using an inkjet spotter (LaboJet-600 Bio) with a nozzle (IJHBS-300) purchased from MICROJET Corporation, Japan. The stage of the inkjet was heated to 60°C to enhance evaporation of the solvent. Three hundred shots (approx. 300 pL/shot) of polymer solutions were dropped onto the membranes of the MSS chip, which was used for the measurements.

### 2.2. Procedure and conditions for the sensing measurements

**Figure 1.**
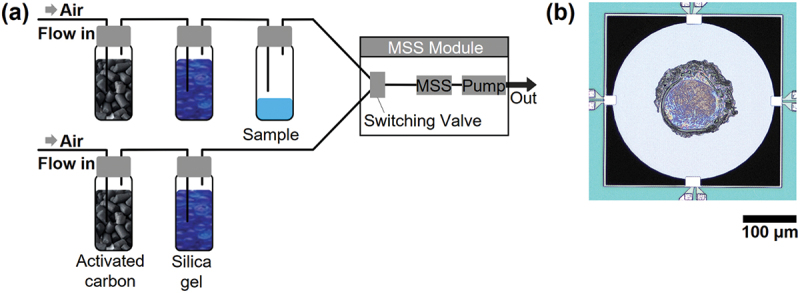
Experimental setup and MSS chip. (a) Schematic of the experimental system used. Air is drawn through two different lines for sampling and purging cycles via a pump and a switching valve. Both lines contain individual vials containing activated carbon and silica gel to provide filtered air. The sample line is drawing the headspace vapour from a vial containing the test chemical in liquid phase. The output signal is recorded by an external computer. (b) Microscope image of the MSS channel coated with a receptor material (PVA).

A schematic illustration of the measurement system is shown in Figure 1a. Before starting the sensing measurements, the MSS channels coated with receptor materials were observed using a laser microscope (VK-X1100, KEYENCE Corporation, Japan); one of the channels is shown in Figure 1b. The sensing measurements of variable flow rates were performed using an MSS standard measurement module (hereinafter denoted as ‘MSS module’) developed by the MSS Alliance [22]. The MSS chips were mounted in a Teflon chamber enclosed with O-rings to form an airtight seal around the MSS chips. The gas flow lines were all composed of Teflon tubes and connectors. The whole system was kept at room temperature. Filtered air was used as a carrier gas, which was prepared by drawing ambient air through activated carbon and silica gel beads. Variable gas flow rates were set by a sequence file, which was read and operated by the MSS module.

**Figure 2.**
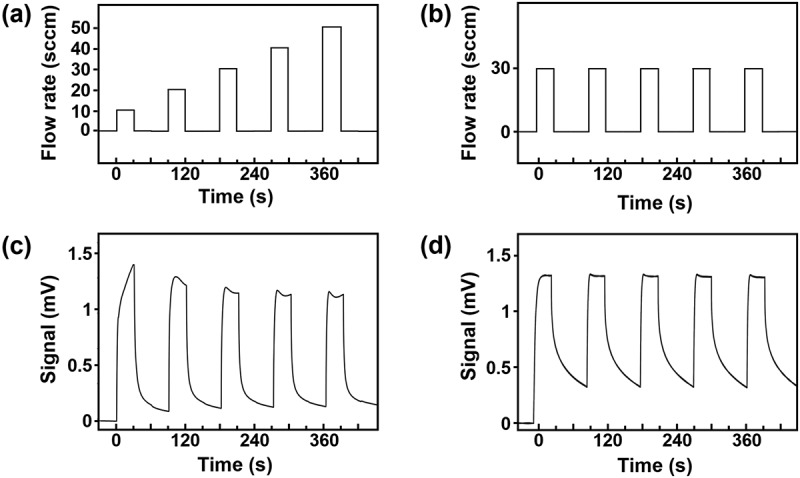
MSS signal response to various gas flow conditions. (a) The gas flow sequence of sampling with various flow rates (from 10 to 50 sccm). (b) The gas flow sequence with a constant flow rate (30 sccm). (c) An example of raw MSS signal response to the gas flow sequence (a). (d) An example of raw MSS signal response to the gas flow sequence (b).

For the sample vapours, headspace gases of liquid samples of cherry, vanilla, lemon, strawberry and lychee (Le Nez du Vin Co., Ltd) contained in glass vials were used. The carrier gas passed through the sample vials, transporting headspace gases to the sensing chamber in the MSS module. To ensure the removal of gases from the system and from the receptor layer, a carrier gas was passed through the measurement system for 300 s prior to each measurement. To reduce transient headspace vapour effects, the system was purged by an initial injection with samples at 50 sccm for 60 s. Subsequently, the pump switched between sampling and purging cycles every 30 s. The flow rate was either varied from 10 to 50 sccm (10, 20, 30, 40 and 50 sccm) or fixed at a certain value. Figure 2a,b present two ways of sensing sequences with different or same flow rates. The sensing sequence was repeated four times for each measurement, and the last three sequences were used for data analysis. For comparison, another set of experiments was performed by repeating the same flow rate in each sequence. In each set of four sequences, nitrogen was flowed for 1 hour to remove remaining samples in the MSS module and the gas flow lines.

### 2.3. Principal component analysis (PCA)

To effectively apply PCA to the signal response data measured by MSS, four feature parameters were extracted from the signal [15,23]:

(1) $$\mathrm{Absorption}\;\mathrm{slope}\;\;:\;\;m_{\mathrm{abs}}=\frac{b-a}{t_{b}-t_{a}},$$

(2) $$\mathrm{Interval}\;\mathrm{slope}\;\;:\;\;m_{\mathrm{int}}=\frac{c-b}{t_{c}-t_{b}},$$

(3) $$\mathrm{Desorption}\;\mathrm{slope}\;\;:\;m_{\mathrm{des}}=\frac{d-c}{t_{d}-t_{c}},$$

(4) $$\mathrm{Intensity}\;\;:\;I=\left(e-a\right),$$

where *a, b, c, d, e, t_a_, t_b_, t_c_* and *t_d_* correspond to signal intensities and time points as denoted in Figure S1. In this case, *t_a_* is defined as the time point at which the sample injection begins for each peak. The subsequent time points, *t_b_* = *t_a_* + 2 [s], *t_c_* = *t_a_* + 30 [s] and *t_d_* = *t_a_* + 32 [s] were selected for detailed analysis. The four parameters were chosen to capture different aspects of the adsorption and desorption processes of sample gas molecules [23,24]. The first parameter reflects the adsorption of gas molecules onto the surface, while the third parameter includes the information about the desorption process. The second parameter is the slope between the adsorption and desorption processes, reflecting the quasi-equilibrium state or viscoelastic characteristics of a receptor material. The fourth parameter, representing the maximum height of the response signal, corresponds to the absorption capacity of the receptor layer. Overall, these parameters were selected to provide a comprehensive understanding of the adsorption and desorption processes.

For the quantitative evaluation of the PCA results, we utilised DBI, which is an index for cluster separation [25,26]. The definition of DBI is given by:

(5) $$\mathrm{DBI}=\frac{1}{n}\overset{n}{\underset{i}{\sum }}\underset{i\neq j}{\max}\left(\frac{\sigma_{i}+\sigma_{j}}{d\left(c_{i},c_{j}\right)}\right),$$

where *n* is the number of clusters, *c*_*k*_ represents the centroid of the cluster *k*. The *σ*_*k*_ variable denotes the mean distance of all elements within the cluster *k* from the centroid of the cluster. The inter-cluster distance between centroids *σ*_*i*_ and *σ*_*j*_ is denoted by *d*(*c*_*i*_,*c*_*j*_). A schematic of DBI is shown in Figure S6. DBI is equal to the sum of the distances of each element in each cluster, divided by the distance between clusters. Thus, a lower value of DBI indicates a larger separation between clusters because of a decrease in the radius of each cluster and/or an increase in the spatial separation between different clusters.

### 2.4. Proton-transfer-reaction time-of-flight mass spectrometry (PTR-TOF-MS)

To measure concentration of each component in odour samples in real-time, proton-transfer-reaction time-of-flight mass spectrometry (PTR-TOF-MS) was performed using PTR-TOF 6000 X 2 (Ionicon Analytik GmbH, Austria). The setup for the experimental measurements is provided in Figure S4. Before injection into the PTR-TOF-MS, the samples were diluted by nitrogen to keep the concentration within the measurable range of PTR-TOF-MS. The detailed calculation of a concentration of each component in the diluted samples is presented in Figure S5 and Table S1. The PTR ion source was operated at 4 mA and 145 V, and the source output voltage was kept at 78.6 V. The drift tube voltage, pressure and temperature were maintained at 557 V, 2.81 mbar and 70°C. The source valve (SV) operating was set at 51%. The ratio between the applied electric field (*E*) and the number density of the gas in the drift tube (*N*) was approximately 101 Townsend (Td; 1 [Td] = 10^−17^ [V cm^2^]). The mass spectrum was recorded in the mass range of 9–400 *m*/*z*. Mass calibration was performed using two ion peaks: hydronium ion isotope (H_3_^18^O^+^, *m*/*z* = 21.022) and diiodobenzene fragment (C_6_H_4_IH^+^, *m*/*z* = 203.943). The count rate of primary ion H_3_O^+^, which was calculated from the count rate at *m*/*z* = 21.022 multiplied by 500, was approximately 1.0 × 10^8^ counts per second (cps) in this work. The temperature in the inlet tube was set at 180°C. The data obtained from PTR-TOF-MS was analysed using PTR-MS Viewer ver. 3.4 and Origin 2021b.

## 3. Results and discussion

### 3.1. Sensing signals by changing flow rates

To observe the variation of sensing signals induced by flow rate, five odour samples were measured as examples at different flow rates ranging from 10 to 50 sccm using an MSS module. As a reference, we also measured at a fixed flow rate (30 sccm) as shown in Figure 2b. Examples of the signal responses of the MSS are shown in Figure 2c,d. It is confirmed that the signal response varies with different gas flow rates (Figure 2c). By contrast, the signal is rather consistent with a fixed flow rate (Figure 2d), while the signal is slightly different in the first cycle, in which the saturated vapour in the initial headspace is injected.

### 3.2. PCA of gas flow effects

**Figure 3.**
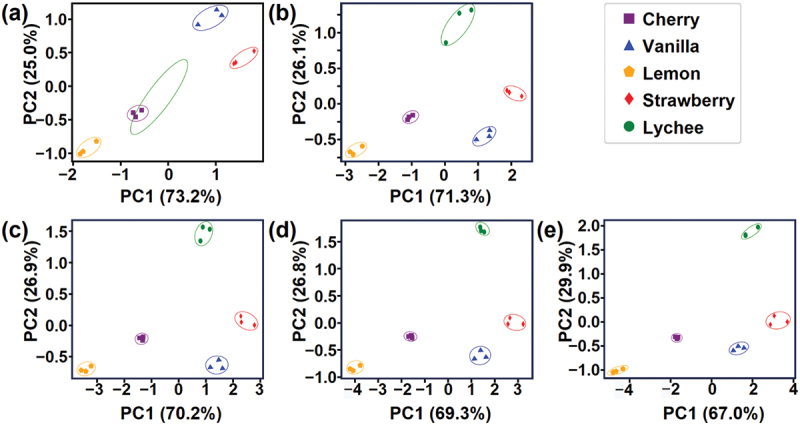
PCA results of five different odours measured using the MSS sensing system. (a)–(e) PCA results with different number of flow rates: one (a), two (b), three (c), four (d) and five (e).

**Figure 4.**
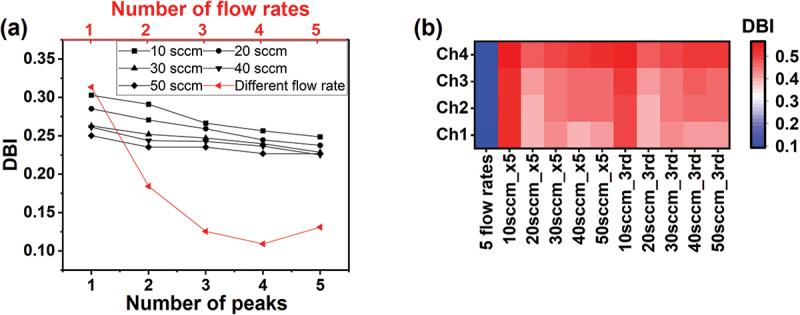
Quantitative evaluation of differentiation performance by gas flow effects. (a) Variation of DBI with different number of measurements. Black symbols correspond to DBI for different number of measurements with fixed flow rates, while red symbols to DBI for different flow rates. (b) Heatmap of DBI of each MSS channel with a different receptor material measured with various flow rates. The DBIs obtained by the measurements with five different flow rates (‘5 flow rates’) are always the lowest for all MSS channels, demonstrating the highest differentiation performance. By contrast, a fixed flow rate with five measurements (‘10sccm_x5’, ‘20sccm_x5’, ‘30sccm_x5’, ‘40sccm_x5’ and ‘50sccm_x5’) and a single measurement at the third injection (‘10sccm_3rd’, ‘20sccm_3rd’, ‘30sccm_3rd’, ‘40sccm_3rd’ and ‘50sccm_3rd’) result in similar DBI values, indicating little contribution of multiple measurements with a fixed flow rate to differentiation performance.

To visualise the effects of flow rate on the sensing signals, we performed PCA by using four features extracted from one sequential measurement. Figure 3 illustrates the results of PCA, where the number of features used for PCA corresponds to the number of flow rates used, specifically 4, 8, 12, 16 and 20 for Figures 3a–e, respectively. In contrast to the overlapped clusters with a single flow rate (Figure 3a), multiple flow rates lead to well-separated clusters (Figure 3b–e). To quantify the differentiation performance with multiple flow rates, DBI was calculated from the results of PCA. As shown in Figure 4a, the DBI decreases significantly as the number of the different flow rates increases, while the decrease in DBI is rather moderate with the fixed flow rates. The DBI was found to decrease significantly at the transition from a single flow rate to two flow rates and get saturated at around three or four different flow rates. Figure S2 shows the PCA results with the loadings of the variables. The most significant change is observed for *m*_int_, particularly in the transition from a single flow rate in Figure S2a to two flow rates in Figure S2b. This is consistent with the relative standard deviation (RSD) trends in Table 1 with *m*_int_ having the highest RSD. The trends of the loadings in the PCA and the parameter values in Table 1 quantitatively illustrate how the introduction of an additional flow rate affects the PCA results.

**Table 1.** Values of four parameters extracted from the signals with different flow rates and corresponding STD, mean and RSD values.

Sample	Parameter	Flow rate (sccm)					STD	mean	RSD
		10	20	30	40	50			(%)
Cherry	*m*_abs_^a^	50.4	66.6	67.5	67.4	64.8	7.3	50.4	11
	*m*_int_^a^	10.6	5.9	4.7	4.4	5.2	2.6	10.6	41
	*m*_des_^a^	−56.3	−61.8	−69.6	−70.2	−66.8	5.9	−56.3	9.0
	*I*^b^	187.5	180.8	178.1	171.4	172.3	6.6	187.5	3.7
Vanilla	*m*_abs_^a^	39.0	58.7	56.5	47.4	47.6	7.9	39.0	16
	*m*_int_^a^	17.9	12.9	13.8	10.3	8.8	3.5	17.9	28
	*m*_des_^a^	−41.1	−46.6	−52.1	−49.2	−45	4.2	−41.1	8.9
	*I*^b^	225.3	226.5	228.5	177.9	176.7	27.1	225.3	13
Lemon	*m*_abs_^a^	47.9	283.4	323.2	333.6	342.4	124	47.9	47
	*m*_int_^a^	59.2	4.4	4.4	8.5	11.6	23.4	59.2	130
	*m*_des_^a^	−30.4	−43.7	−56.2	−54.8	−55.1	11.1	−48.1	23
	*I*^b^	1.1	1.6	1.3	2.2	1.1	0.5	1.1	32
Lychee	*m*_abs_^a^	30.1	40.8	44.4	45.4	46.4	6.7	30.1	16
	*m*_int_^a^	8.7	6.1	5.0	4.5	4.5	1.8	8.7	31
	*m*_des_^a^	−28.9	−36.2	−37.5	−38.5	−38.5	4.1	−28.9	11
	*I*^b^	130.3	131.1	132.3	128.8	130	1.3	130.3	1.0
Strawberry	*m*_abs_^a^	21.6	28	28.8	30.9	30.2	3.7	21.6	13
	*m*_int_^a^	7.3	5.1	4.6	4.3	4.2	1.3	7.3	25
	*m*_des_^a^	−20.8	−27.0	−30.6	−30.6	−30.2	4.2	−20.8	15
	*I*^b^	105.0	97.3	97.7	101.0	95.8	3.7	105	3.7

^a^Parameters in the unit of µV/s.

^b^Parameters in the unit of µV.

Figure S3 provides further insight into how the different flow rates affect the PCA results. Figure S3a compares the DBI for each peak at the same flow rate, plotted in black, and multiple peaks with different flow rates, plotted in red. It should be noted that the number of parameters used for the PCA is always 4 for the black plots and 4, 8, 12, 16 and 20 for the red plots with the number of flow rates of 1, 2, 3, 4 and 5. This result indicates that all peaks have a similar amount of information when the flow rate is kept constant, as shown by similar DBI values in the black plots, while the combination of different flow rates improves the differentiation performance as demonstrated by low DBI values plotted in red. Meanwhile, Figure S3b (same as Figure 4a) compares the DBI for different numbers of peaks with the same flow rate plotted in black and those obtained with different flow rates plotted in red. It should be noted that the number of parameters used for the PCA (and the DBI evaluation) is 4, 8, 12, 16 and 20 with the number of peaks being 1, 2, 3, 4 and 5 for both the black and red plots. This result demonstrates that simply increasing the number of peaks (and thus the features for PCA) does not have enough impact on the differentiation performance when the flow rate is kept constant, as shown with similar DBI values in the black plots. By contrast, the combination of different flow rates improves the differentiation performance, as demonstrated with low DBI values plotted in red, similar to Figure S3a. These results highlight the critical importance of the variation introduced by different flow rates in the differentiation of complex odour samples.

Figure 4b shows a heatmap of DBI for each MSS channel with a different receptor material measured with various flow rates. For all MSS channels, the lowest DBIs were observed with the five different flow rates. This consistency across channels indicates that the current methodology is applicable to various receptor materials. It highlights the potential of this approach to reduce the need for multiple receptor materials while maintaining differentiation accuracy, thus simplifying sensor design without compromising performance.

### 3.3. Gas flow effects on sensing signal

We further investigated the gas flow effects on sensing responses. To illustrate the trends of each parameter with various flow rates, the variation of each parameter was evaluated by calculating RSD as shown in Table 1. Among the four parameters, the Interval slope (*m*_int_) consistently shows the highest RSD values, indicating that the variation in flow rate has a significant effect on the Interval slope. Since RSD is calculated by dividing a standard deviation (STD) by a mean value, a smaller mean value will result in a larger RSD. Such small mean values are frequently observed for the Interval slope, because Interval slope can have either positive or negative values, which cancel each other, resulting in smaller mean values. The negative values of the Interval slope are observed when the sensing signals show overshoot during the adsorption of gas molecules on the receptor layers. The appearance of such overshoot behaviour could be attributed to the following three reasons: (1) viscoelastic property of receptor materials, (2) chromatographic effect of gas flow line and (3) initial vapour in the headspace. For the viscoelastic property, it was confirmed by the analytical model that the balance between three parameters (diffusion time constant of gas molecules into the receptor layer, relaxation time constant of the receptor layer and the ratio of Young’s moduli at unrelaxed and relaxed states of the receptor layer) determines the shape of sensing signals for nanomechanical sensors including MSS [24,27]. For example, with a certain ratio of unrelaxed and relaxed moduli, overshoot appears when the diffusion time constant is smaller than the relaxation time constant. Such a condition is frequently observed in various combinations of receptor materials and gas molecules. Moreover, the adsorption of gas molecules could induce modulations of viscoelastic behaviours of the receptor materials. The chromatographic effect is observed in the Teflon tube connecting between the sample vial and sensor, since Teflon absorbs some amount of molecules even though it is known to be inert [28]. There should be a difference in the retention time of each component, resulting in large variations in the Interval slope and in the Absorption slope. As for the initial vapour in the headspace, it is correlated with the evaporation rate of each component. Since the injection of carrier gas into the headspace provides vapours with high concentrations of components evaporated during the purging process, such vapours could induce abrupt increase in the sensing signal, resulting in overshoot behaviour in some cases.

The trends of other parameters could also explain some behaviours in sensing signals and the mechanisms involved. For the Absorption slope and Desorption slope, it is confirmed that the RSD of the former parameter is almost always larger than that for the latter one. The main reason for this trend could be attributed to the fact that the adsorption process involves both evaporation of sample liquid and adsorption of sample gas molecules into the receptor material, whereas there is only desorption of the samples from the receptor materials in the desorption process. Since the flow rates affect the evaporation of sample liquid, the measurements at different flow rates provide additional variations in the Absorption slope. The RSDs of intensity are rather small compared to RSDs of other parameters. This trend could be also explained partially by the analytical model of the viscoelastic behaviour in nanomechanical sensing. According to the analytical model, the signal intensity is mainly determined only by the analyte concentrations [29,30], which do not usually exhibit complex dynamic behaviours, especially in the equilibrated state after the transient state during the adsorption process. This result suggests that simple extraction of signal intensity does not provide sufficient information for the differentiation of samples.

### 3.4. PTR-TOF-MS analysis of gas flow effects

**Figure 5.**
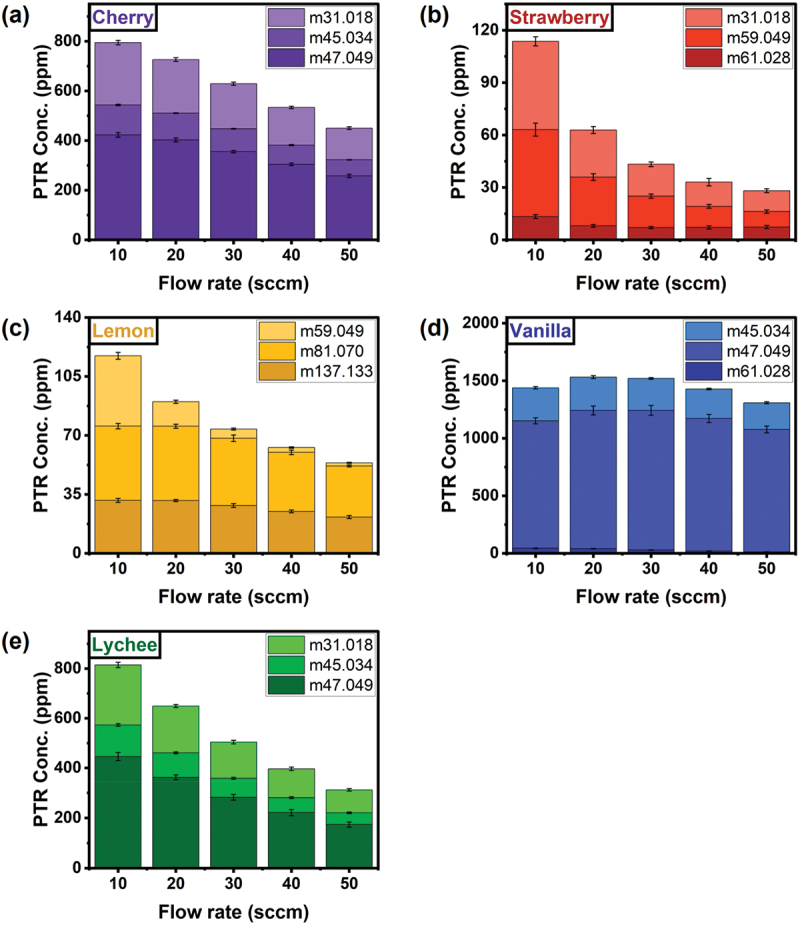
Evaluation of the gas flow effects on the concentrations of major components by PTR-TOF-MS. (a)–(e) real-time concentrations of the three main components of (a) cherry, (b) strawberry, (c) lemon, (d) vanilla and (e) lychee odours.

Complex odours are usually composed of multiple volatile components. Variation of the flow rate of the carrier gas affects the concentrations of the components. To observe and clarify such variation of concentrations, the concentrations of the components in each sample gas were measured by PTR-TOF-MS in real-time. The concentrations of the three major components in five odour samples with different flow rates are shown in Figure 5. Since the concentration of the saturated vapour is too high to be measured by the PTR-TOF-MS, nitrogen was introduced to dilute the sample vapour (see Section 2.4). Figure S5 shows one example of raw real-time concentration data from PTR and dilution formula for the gas sample connected to PTR. The dilution rates to each flow rate are summarized in Table S1. It is confirmed that the concentrations of each component tend to decrease as the gas flow rate increases from 10 to 50 sccm. The major components were found to be similar in these samples most probably because of the similar solvents used in these samples. Nevertheless, the trends of concentration variations are different in each sample, since the compositions of components with various concentrations should affect the volatility of components in different ways. Some samples have different viscosity, which should also affect the volatility of the components. Such dependence of the concentration of each component on the flow rate can be attributed to the larger separation in PCA with multiple flow rates with lower DBI. To verify if the different sequence of the flow rates affects these concentration trends, the concentration of each component in the samples was measured in the sequences starting from high flow rate (50 sccm) to low flow rate (10 sccm) as well as in a random flow rate sequence. The results of the PTR-TOF-MS show nearly identical trends in each sequence, indicating that the sequence of flow rates has an almost negligible effect on the concentrations of the volatile components.

## 4. Conclusion

In this study, we proposed a new system-based method to augment sensing signals in olfactory sensors by using different gas flow rates that provide additional information for better discrimination of complex odour samples. We quantitatively evaluated the effect of this method through PCA and DBI of the sensing signals of complex odour samples. The analysis of the RSD of the parameters extracted from the sensing signals gives insight into the related factors, such as evaporation of liquid odour samples, gas/solid equilibrium and chromatographic effects in the gas flow lines. The real-time gas analyses with PTR-TOF-MS confirm that the concentrations of each component in the odour samples vary with different flow rates. This observation is consistent with the trend observed in the output signals of the MSS. Since the modulation of gas flow rate induces various effects, the presented approach may also provide physical/chemical insight into sample properties and/or sorption kinetics. It should be noted that this signal augmentation method can be generally applied to various types of gas sensors and provide more information on complex odour samples. This approach expands the applicability of olfactory sensors in biomedical, chemical, agricultural and food safety fields through the simple and effective signal augmentation.
